# Objective assessment of health or pre-chronic disease state based on a health test index derived from routinely measured clinical laboratory parameters

**DOI:** 10.1186/s12967-015-0487-z

**Published:** 2015-04-22

**Authors:** Sun Wenping, Liu Ying, Leng Song, Li Yuzhong, Liu Hui

**Affiliations:** College of Medical Laboratory, Dalian Medical University, Dalian, 116044 China; Second Affiliated Hospital of Dalian Medical University, Dalian, China

**Keywords:** Health evaluation, Laboratory medicine, Biomarker, Ill-health

## Abstract

**Objective:**

To develop a quantitative system to enable the objective assessment of health or pre-chronic disease state.

**Methods:**

On the basis of measured values and reference ranges, we obtained the organ function index (mean of the cut-off ratios of albumin and creatinine), blood lipid index (mean of the cut-off ratios of triglycerides, cholesterol, high-density lipoproteins and low-density lipoproteins), stress index (mean of the cut-off ratios of neutrophils and glucose), and the health test index (mean of the above three indexes, HTI). Elderly populations, individuals with nonalcoholic fatty liver disease and administrators were included in the groups of observed subjects to verify the organ function index, blood lipid index and stress index.

**Results:**

The scores of the three indexes were all statistically higher in the observed group than in the control group (*p* < 0.05). The mean HTI score was 0.7 ± 0.07 and was normally distributed in the control population. The rates of hypertension, obesity, fatty liver disease and health (undetectable organic diseases) increased with increasing HTI scores in a random population.

**Conclusions:**

The HTI is easily derived from routinely measured clinical laboratory parameters. It can reflect the health status of an individual and may be a useful tool for the quantitative differentiation of health status.

## Introduction

In modern society, people are highly concerned about their health. This is manifested by an increasing number of people who participate in health examinations and monitor their health status. General health examinations assess health status by checking for diseases, and it is very difficult to quantitatively assess health levels with these more subjective qualitative techniques. We hypothesise that health status can be described as a quantitative event, and a poorer health status is associated with a higher probability of developing disease.

Health status is influenced by social, psychological and biological factors [1-3]. Hence, developing a quantitative evaluation of health status is highly challenging. Typically, clinical data are derived from informal, subjective methods, such as questionnaire surveys, which are highly influenced by subjective factors. They also require the cooperation of subjects and are time-consuming to complete and analyse, meaning that this approach cannot be easily applied. Therefore, a new method of quantitative evaluation is required to assess general health status.

Deteriorations in health status or pre-chronic disease state caused by any reason will induce changes in a range of biological factors that increase the possibility of developing disease [4,5]. This process is accompanied by changes in clinical laboratory parameters [6-8]. Therefore, clinical laboratory parameters could be used to evaluate health status. In modern society, health status is mainly influenced by psychosocial stress, overnutrition and the ageing process, whereby, three group indicators, such as the neutrophil count and glucose level (reflecting stress) [9-11]; the triglyceride, cholesterol, high-density lipoprotein and low-density lipoprotein levels (reflecting blood lipids) [12-14]; and the albumin and creatinine values (reflecting liver and kidney function) [15-17] can be used to evaluate health status with respect to three dimensions. Our group has used laboratory parameters to evaluate health status with respect to one dimension [18,19], and this may be the first report to establish an index for evaluating health status with regard to multiple dimensions using laboratory parameters.

This study was designed to develop a reliable method to undertake multiple measurements of routinely measured laboratory indices that are associated with health status. The basic concept underlying the analysis was the evaluation of the impact of different trait-based health indices of somatic health as indictors of health status, which can be used to simplify a complex problem. Dynamic changes in adverse health status-related experimental indicators within specific ranges can be observed systematically, and biological markers of health status can be selected. In this manner, we aimed to establish a quantitative or semi-quantitative system to objectively assess the general health levels of an individual using laboratory indices.

## Methods

### Evaluation indicators and evaluation model

The evaluation indicators of organ function included albumin (Alb, 37 ~ 53 g/L) and creatinine (CRE, 60 ~ 130 μmol/L for males; 40 ~ 110 μmol/L for females); the evaluation indicators of blood lipids included triglycerides (TG, 0.22 ~ 2.29 mmol/L), cholesterol (Chol, 2.80 ~ 5.20 mmol/L), high-density lipoprotein (HDL, 0.80 ~ 3.26 mmol/L) and low-density lipoprotein levels (LDL, <3.12 mmol/L); and the evaluation indicators of stress included the neutrophil count (Neut; 2.0 ~ 7.0× 10^9^/L) and glucose level (GLU: 3.60 ~ 6.10 mmol/L).

The cut-off values could be obtained from the upper or lower limits of the reference range on the basis of the clinical significance of each indicator. According to the cut-off values, the above indicators were converted into a ratio of cut-off values. A larger cut-off ratio represented a worse health status, and the numerators and denominators of the measured values of various indicators that were converted into the cut-off ratios were different. For ALB and HDL, the lower limit value was recorded as the numerator, and the measured value was recorded as the denominator. For the other indicators, the upper limit value was recorded as the denominator, and the measured value was recorded as the numerator. The cut-off values and formula for calculating the ratio of cut-off values for each indicator is listed in Table 1.

**Table 1:** **The cut-off values and formula for calculating the ratio of cut-offs for each indicator**

**Indicators**	**Cut-off values**	**Ratio of cut-offs**	**Indicators**	**Cut-off values**	**Ratio of cut-offs**
Alb	37	37/MV	TG	2.29	MV/2.29
CRE	Male: 130; Female: 110	Male: MV/130; Female: MV/110	Chol	5.20	MV/5.20
Neut	7.0	MV/7.0	HDL	0.80	0.80/MV
Glucose	6.1	MV/6.1	LDL	3.12	MV/3.12

MV: measured value.

For example, if the upper limit value of Neut was 7.0, and the measured value of Neut was 3.5, the ratio of the cut-off value was 0.5 (3.5/7.0) for Neut. In theory, an HTI score >1 was considered a marker of disease.

The means of the cut-off ratios of ALB and CRE were defined as the organ function index, whereas the means of the cut-off ratios of TG, Chol, HDL and LDL were defined as the blood lipid index, and the means of the cut-off ratios of Neut and GLU were defined as the stress index. Finally, the mean of the above three indexes was defined as the health test index (HTI).

### Subject selection

All subjects were Chinese individuals who had visited the Second Affiliated Hospital of Dalian Medical University, China, for routine health check-up examinations. The experiments were conducted in accordance with the Declaration of Helsinki. The blood samples were taken during routine care of the subjects, rather than for research purposes alone. The Institutional Ethics Committee of Dalian Medical University approved the study and waived the need for written informed consent from the participants due to the observational nature of the study.

An elderly population was examined to verify the organ function index [19]. The elderly population consisted of 100 individuals (50 males and 50 females) over 50 years of age without organic disease as detected by imaging examinations. Their average age was 61.3 ± 10.1 years. The control group consisted of 300 individuals (150 males and 150 females) between 20 and 50 years of age without organic disease as detected by imaging examinations with an average age of 30.2 ± 4.7 years.

Individuals with nonalcoholic fatty liver disease were examined to verify the blood lipid index [20]. Fatty liver diagnoses were made using ultrasonography based on the finding of a bright liver (increased echogenicity) with liver − kidney contrast (increased echogenicity of the liver compared to the right kidney). Subjects in the fatty liver group who met the following criteria were excluded from the study: (1) those suffering from other liver diseases, such as viral hepatitis; (2) a dependence on alcohol; and (3) >40 years of age. The fatty liver group consisted of 100 patients (50 males and 50 females) with an average age of 32.3 ± 4.6 years. The control group consisted of 200 individuals (100 males and 100 females), 20 to 40 years of age without organic disease according to imaging examinations, with an average age of 30.7 ± 4.8 years.

Administrators were examined to verify the stress index [21]. A total of 36 administrators (25 males and 11 females) were selected from the deans of a University in China. All administrative work was performed by themselves without the assistance of their secretaries. Their average age was 45.2 ± 4.3 years. The control group consisted of a total of 36 teachers with an academic title below associate professor and without an administrative post at the same school, and their age (43.6 ± 4.2 years) and sex (25 males and 11 females) were similar to the administrative management group.

The healthy population was examined to test the validity of the HTI. A total of 400 subjects (200 males and 200 females) without organic disease as confirmed by physical examination were included as subjects with an average age of 37.9 ± 15.0 years.

In addition, a random population was examined to observe the relationship between the HTI and the health status. A total of 1,200 individuals (600 males and 600 females) were selected from the university staff with an average age of 46.4 ± 16.3 years.

### Blood sampling and blood analyses

At 0700, fasting blood samples (5 mL) were drawn from the antecubital vein of the subjects in two vacuum tubes, one of which contained an anticoagulant and was used for blood cell counts, and the other was used for the determination of biochemical indices. To obtain serum, the tubes were centrifuged for 15 min. After separation, the serum samples were stored at −70°C until further analysis.

For each evaluation, the neutrophil count was determined using an automated haematology analyser (Sysmex 2100; Japan). Serum samples were thawed thoroughly at room temperature and subsequently assayed in same run. The biochemical indices were measured by an automatic biochemistry analyser (Hitachi 7600). The haematological and biochemical investigations were performed in the clinical laboratory of our University Hospital using standard commercial reagent kits.

### Imaging examinations

The imaging examinations included ultrasonography and X-ray examinations. Ultrasonography was conducted with a Hitachi 7500 and an ALOKA3500 diasonograph with a probe frequency that ranged from 3.5 to 5 MHz. The examined organs included the liver, gallbladder, spleen, pancreas, kidney, prostate (males) and uterus and its appendages (females). Before the examinations, all subjects fasted for over 8 h. The abdominal examinations were conducted with the subjects in a supine position and in the left and right lateral positions. The subjects drank 1–1.5 L water within 1–2 h before the ultrasound examination of the bladder, prostate (males) and uterus and its appendages (females) to ensure that the bladder was filled.

The chest X-ray examinations were conducted with a Shimadzu X-ray diagnostic apparatus (SHIMADZU HL150). Anteroposterior and lateral chest radiographs were acquired during the resting stage of deep inspiration.

Various pathological changes or suspected pathological changes in the above imaging reports, except for skeletal abnormalities, were defined as organic lesions in this study. Subjects without any specific imaging findings, except for skeletal abnormalities, were considered ‘normal’ in this study.

### BMI and hypertension

The body mass index (BMI) was calculated as the subject’s weight (kg) divided by their height (m) squared. A BMI *≥*25.0 was defined as obesity according to the classification of adult body weight in Asian populations [22].

The blood pressure was measured twice consecutively with a standard mercury sphygmomanometer according to the 1999 guidelines of the World Health Organization and the International Society of Hypertension (WHO/ISH) [23]. Subjects with a systolic pressure ≥140 mmHg and/or a diastolic pressure ≥90 mmHg in the absence of antihypertensive drugs were considered hypertensive.

### Statistical analysis

Using the measurement values and reference ranges, we obtained the organ function index, blood lipid index, stress index, and HTI as described in the “Evaluation indicators and evaluation model” section. The indices were described in terms of quartile values because some of the indices had a non-normal distribution. The Mann–Whitney *U*-test was used to analyse the differences among different groups.

A Normal P-P Plot of the HTI was constructed to determine whether the HTI showed a normal distribution in a healthy population.

Statistical analyses were performed with SPSS statistical analysis software for Windows (SPSS, Chicago, IL, USA). A difference was considered statistically significant when the P-value was less than 0.05 (two-tailed test).

## Results

**Figure 1:**
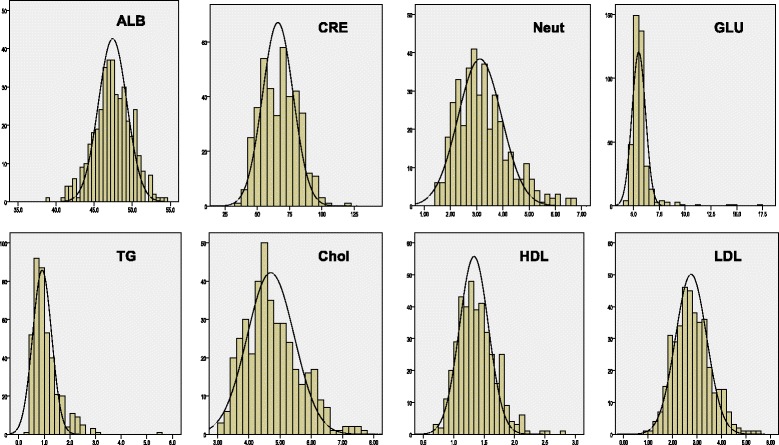
The distribution of the original data in the healthy population.

The distribution of the original data in the healthy population is shown in Figure 1. Some of them exhibit non-normal distributions.

The original data for the organ function index, blood lipid index and stress index for the different observed groups are shown in Tables 2, 3 and 4. The scores of the three indexes were all significantly higher in the observed group than in the control group (*p* < 0.05).

**Table 2:** **Comparison of the organ function index between the elderly and control populations**

**Group**	**ALB 50** ^**th**^ **(25th, 75th)**	**CRE 50** ^**th**^ **(25th, 75th)**	**Function Index 50** ^**th**^ **(25th, 75th)**
Old	46.15 (44.30, 47.38)	69.00 (59.00, 79.00)	0.69 (0.65, 0.74)
Control	47.80 (46.43, 49.30)	67.00 (54.00, 76.75)	0.66 (0.63, 0.69)
*Z*	6.636	2.133	5.291
*P*	<0.0001	0.034	<0.0001

**Table 3:** **Comparison of the blood lipid index between the fatty liver disease and control populations**

**Group**	**TG 50** ^**th**^ **(25th, 75th)**	**Chol 50** ^**th**^ **(25th, 75th)**	**HDL 50** ^**th**^ **(25th, 75th)**	**LDL 50** ^**th**^ **(25th, 75th)**	**Lipid Index 50** ^**th**^ **(25th, 75th)**
Fatty liver	1.85 (1.24, 2.39)	4.96 (4.45, 5.66)	1.09 (0.98, 1.26)	3.03 (2.55, 3.51)	0.87 (0.78, 1.00)
Control	0.86 (0.66, 1.10)	4.35 (3.92, 4.88)	1.34 (1.12, 1.58)	2.54 (2.11, 3.04)	0.67 (0.60, 0.75)
*Z*	9.440	5.282	5.189	4.342	9.038
*P*	<0.0001	<0.0001	<0.0001	<0.0001	<0.0001

**Table 4:** **Comparison of the stress index results between the administrators and the control population**

**Group**	**Neut 50** ^**th**^ **(25th, 75th)**	**GLU 50** ^**th**^ **(25th, 75th)**	**Stress Index 50** ^**th**^ **(25th, 75th)**
Dean	2.95 (2.49, 3.80)	5.90 (5.49, 6.22)	0.71 (0.65, 0.79)
Control	2.64 (2.14, 3.65)	5.64 (5.39, 5.97)	0.66 (0.60, 0.72)
*Z*	1.594	2.033	2.219
*p*	0.111	0.042	0.027

**Figure 2:**
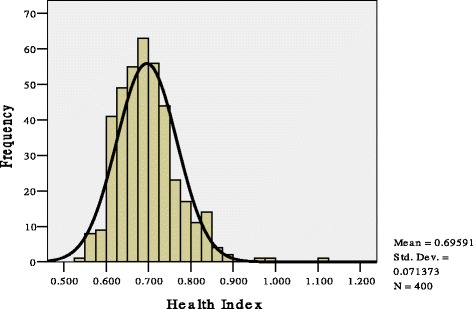
The distribution of the health test index (HTI) results in a healthy population.

**Figure 3:**
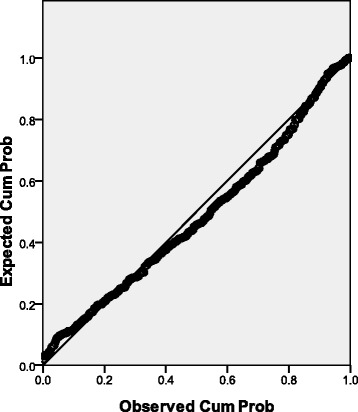
Normal P-P Plot of the health test index (HTI).

The distribution of the HTI in the healthy population is shown in Figure 2. The mean was 0.70, and the standard deviation was 0.07. A linear relationship between the expected and observed variables was found in the Normal P-P Plot (Figure 3), suggesting a normal distribution of the HTI.

The HTI scores were measured in a population containing 1,200 randomly selected individuals. The HTI scores were ordered from the smallest to the largest values and divided into lower, medium and higher groups containing 400 individuals. The rates of hypertension, obesity, fatty liver disease and health (undetectable organic diseases) in the various groups are shown in Table 5.

**Table 5:** **Incidence rates of hypertension, obesity, fatty liver disease and normality (no detectable organic diseases) in 1,200 randomly selected individuals**

**HTI group**	**Health test index (HTI)**		**Incidence (%)**			
	**Range**	**Mean**	**Hypertension**	**Overweight**	**Fatty liver**	**Normality**
Lowest 400	0.537~	0.64	9.9	11.4	3.7	51.8
Middle 400	0.685~	0.72	31.6	35.3	30.0	34.5
Highest 400	0.765 ~ 1.753	0.84	58.5	53.3	66.3	13.7

## Discussion

Most laboratory indicators reflect the risk of a disease rather than the presence of a disease; therefore, although laboratory indices are mainly used for the diagnosis of disease, they are considered indicators of health or pre-chronic disease state [18,24,25]. The changes of laboratory indicators could be obtained efore organic disease, therefore, non-normal distributions could be observed for some laboratory indicators in subjects without organic disease. In the present study, experimental indicators are divided into three groups (organ function, blood lipid levels and stress status) to evaluate the health status with respect to three dimensions (aging, blood lipids and stress status) and reflect the influence of different factors on health. According to the organ function, blood lipid level and stress index scores, the related indexes can respond to predetermined factors that affect health, which indicate that the experimental indicator selection is reasonable.

It is very difficult to accurately determine the standard deviation of each indicator because the standard deviation values use different measurement systems, and some indicators are not normally distributed; therefore, the standard deviation values in various laboratories are different. If a less accurate standard deviation is introduced into the evaluation model, uncertainty factors are added. However, although the standard deviation and reference ranges of experimental indicators provided by different clinical laboratories are different, each laboratory will pay close attention to the upper or lower limits of the reference range. Therefore, the upper limit or lower limit of the reference ranges were considered the reference criteria in the process of comparing different indicators, and it is also beneficial to the compare measured results between different laboratories. Our study used the cut-off ratio as indicators to simplify the calculations, and the study results confirmed that this method of processing data ensured accurate results, which means that these index scores derived from the ratio values correspond to different health statuses.

In theory, an HTI score >1 is considered a marker of disease. In the healthy population in our study, the mean of the actual observed HTI was 0.70, and the standard deviation was 0.07; therefore, it was considered a one-tailed test distribution; if the HTI was >0.8 (0.7 + 1.64*0.07), it was considered to indicate ill health or pre-chronic disease state, and medical investigations were recommended. Put simply, a lower HTI corresponded to a better health status.

As there is no gold standard of ill health, we detected the experimental health indices of 1,200 randomly selected individuals and found that a higher HTI indicated a worse health status and a higher the probability of organic diseases (Table 5). Therefore, we suggest that the HTI can reflect the health status and is a valid biological marker of health.

However, some limitations of the study should be noted. The indexes were generated by the mean of the multiple indicators in the present study. Although the mechanisms are different for increases or decreases of each indicator, it has not been determined whether each parameter is distributed independently. Our previous work suggested that biological variations are independent of each other for most laboratory parameters [26], thus, it is beneficial to avoid the impact of biological variations when evaluating health with using the multiple laboratory parameters.

## Conclusions

Various factors influence health through biological pathways and could be detected by small changes in the HTI before the presentation of clinical symptoms, and these small changes may be the only information that can be obtained in the pre-disease state. Moreover, the HTI score is objective and quantitative in the evaluation of physical health, does not require the cooperation of subjects and can be used for the cross-temporal comparison of health status between different populations. Therefore, the HTI derived from routinely measured clinical laboratory parameters could become a powerful tool for the evaluation of health or pre-chronic disease state.

## Abbreviations

Alb: Albumin
BMI: Body mass index
Chol: Cholesterol
CRE: Creatinine
GLU: Glucose
HDL: High-density lipoprotein
HTI: Health test index
LDL: Low-density lipoprotein
Neut: Neutrophil count
TG: Triglycerides
